# Supplementary material for: Measures of skin conductance and heart rate in alcoholic men and women during memory performance
Source: PeerJ. 2015 May 5;3:e941. doi: 10.7717/peerj.941 (PMC4435500; doi:10.7717/peerj.941)
Supplement: Table S1 — Means and standard deviations (SD) are displayed for age, education, measures of drinking history, the Wechsler Adult Intelligence Scale (WAIS) and the Wechsler Memory Scale (WMS). Besides amount and duration of drinking, these characteristics did not differ significantly between the ALC and NC groups. [file peerj-03-941-s002.docx]

|  | **Alcoholic Participants** | | | | | |
| --- | --- | --- | --- | --- | --- | --- |
|  | **All (N = 24)** | | **Women (N = 10)** | | **Men (N = 14)** | |
|  | **Mean** | **SD** | **Mean** | **SD** | **Mean** | **SD** |
| Age (years) | 55.6 | 10.0 | 57.3 | 9.5 | 54.4 | 10.6 |
| Education (years) | 15.0 | 2.2 | 16.4 | 1.8 | 13.9 | 1.9 |
| Duration of Heavy Drinking (years) | 16.0 | 5.8 | 14.2 | 2.6 | 17.4 | 7.1 |
| Daily Drinks | 13.3 | 9.8 | 12.4 | 9.2 | 13.9 | 10.5 |
| Length of Sobriety (years) | 7.7 | 11.0 | 9.6 | 12.5 | 6.3 | 10.1 |
| WAIS-III Full Scale IQ | 108.1 | 14.4 | 109.3 | 12.9 | 107.2 | 15.7 |
| WAIS-III Verbal IQ | 110.8 | 12.3 | 112.2 | 11.2 | 109.9 | 13.3 |
| WAIS-III Performance IQ | 102.9 | 16.9 | 104.1 | 17.4 | 102.0 | 17.1 |
| WMS-III Immediate Memory | 107.0 | 18.0 | 110.9 | 22.5 | 104.1 | 14.2 |
| WMS-III Delayed Memory | 109.5 | 19.4 | 113.5 | 26.9 | 106.7 | 11.9 |
| WMS-III Working Memory | 102.8 | 11.5 | 105.0 | 10.6 | 101.3 | 12.2 |
|  |  |  |  |  |  |  |
| **Nonalcoholic Participants** | | | | | |  |
|  | **All (N = 21)** | | **Women (N = 11)** | | **Men (N = 10)** | |
|  | **Mean** | **SD** | **Mean** | **SD** | **Mean** | **SD** |
| Age (years) | 52.1 | 14.1 | 56.1 | 16.0 | 47.8 | 11.0 |
| Education (years) | 15.8 | 1.8 | 15.8 | 1.7 | 15.8 | 2.0 |
| Duration of Heavy Drinking (years) | 0.2 | 0.6 | 0.0 | 0.0 | 0.4 | 0.8 |
| Daily Drinks | 0.4 | 0.6 | 0.4 | 0.7 | 0.4 | 0.5 |
| Length of Sobriety (years) | 3.0 | 7.7 | 2.8 | 8.8 | 3.2 | 6.6 |
| WAIS-III Full Scale IQ | 112.4 | 13.1 | 116.9 | 13.8 | 107.4 | 10.8 |
| WAIS-III Verbal IQ | 112.9 | 13.1 | 117.1 | 13.6 | 108.3 | 11.3 |
| WAIS-III Performance IQ | 109.6 | 14.5 | 113.4 | 13.2 | 105.5 | 15.3 |
| WMS-III Immediate Memory | 111.9 | 16.6 | 119.8 | 13.9 | 103.1 | 15.3 |
| WMS-III Delayed Memory | 110.8 | 13.4 | 116.7 | 11.1 | 104.3 | 13.1 |
| WMS-III Working Memory | 106.7 | 12.0 | 110.4 | 10.9 | 102.6 | 12.2 |
